# Contributions from Approved Societies to Hospitals

**Published:** 1913-11-15

**Authors:** 


### NATIONAL INSURANCE ACT DEPARTMENT.

### LXXIII.—Contributions from Approved Societies to Hospitals.

Since the article on "The Effect of the Amendment Act on Hospital Finance," which appeared in our issue of November 1, was written a very important circular has been issued by the Insurance Commissioners to the approved societies bearing on the question of their contributions to hospitals. In our issue of November 1 we were dealing with the subject entirely from the point of view of Section 12 of the principal Act, as amended by Section 15 of the Act of 1913, which refers to the possible application of sickness benefit for members without dependants while in hospital towards their maintenance and treatment under agreement between the approved societies and hospitals concerned.

The circular now issued by the Insurance Commissioners deals with the larger issue, and indicates their attitude to the powers conferred on approved societies under Section 21 of the principal Act of contributing to hospitals.

The matter is of such considerable interest to our readers that we venture to print the circular in full as follows:—

### The Insurance Commissioners' Circular.

The Insurance Commissioners have been asked for information as to the powers of approved societies under Section 21 of the National Insurance Act, 1911.

That section enables a society to make such subscriptions or donations as it may think fit to hospitals, dispensaries, or district nursing associations, and to appoint nurses for the purpose of visiting and nursing insured members of the society.

The section also provides that any sums so expended by a society shall be treated as expenditure on such benefits as may be prescribed.

The Commissioners propose to make regulations at an early date providing that expenditure by approved societies under the section shall be treated as expenditure on sickness benefit. The State grant (so far as the expenditure relates to British subjects) will be payable in respect of any sums properly expended by a society under the section.

Societies should, however, understand clearly that subscriptions or donations of the kind contemplated by Section 21 of the Act must come from their ordinary benefit funds, and that no margin has been provided in those funds specially for the purpose of meeting expenditure under the section. It is for a society to decide for itself whether judicious expenditure of this kind, in return for which members of the society will be able to obtain skilled attendance in illness, may be expected to have the effect of promoting the recovery of sick members, and so relieving the ordinary expenditure on sickness benefit.

As, however, such relief might not be experienced immediately, societies, before incurring expenditure under the section, will be well advised to have regard to their immediate liabilities for expenditure out of their sickness benefit funds, and may think it desirable to obtain actuarial advice on the subject. In the first instance, therefore, societies may think it desirable not to incur expenditure under Section 21 unless they have already realised actual savings (sufficient to cover the expenditure) by carrying to the administration account less than the maximum amount which is available for administration under the regulations. Subsequent experience will show whether further expenditure can safely be made, and whether a society may reasonably hope to receive an adequate return for such expenditure in the form of a decrease in ordinary expenditure on sickness benefit.

Any sums so expended should be entered in the cash-book as "subscriptions or donations to hospitals, etc. (Section 21)," and should be carried to a separate account under the same title in the ledger.

As regards expenditure on nurses appointed for the purpose of visiting and nursing insured members, it should be understood that such expenditure can only be treated as expenditure on benefits under Section 21 of the Act if the persons are appointed for actual sick-nursing duties. The payment of sick visitors whose duties are purely of an administrative character, and do not include sick nursing, cannot be met under Section 21, but must be charged to the administration account.

The payment of midwives also stands on a different footing, inasmuch as maternity benefit may be administered otherwise than in cash, and the society is therefore entitled to place at the disposal of its members or their wives the services of duly certified midwives. In accordance with the arrangements explained in Circular A.S. 73, the payment of such midwives would be made out of the sum payable as maternity benefit to the individual members.

### Points to be Noted.

There are a few points which obviously call for comment. In the first place, it should be noted that the circular has reference to powers conferred by the Act of 1911, and, although that Act has been in actual operation for more than fifteen months and benefits have been paid for nearly ten months, the Insurance Commissioners have only just disclosed their intention in the matter. They have not even yet issued the formal regulations entitling the approved societies to make the contributions indicated, and until this has been done the societies will remain powerless to act in the matter. Although belated in its appearance the circular will doubtless be welcomed as an evidence of the intention of the Insurance Commissioners to give greater prominence in future to the work of the hospitals in the administration of health insurance.

### The Funds that are to be Available.

In the second place, it is rather curious to observe the non-committal attitude of the Insurance Commissioners. For while it is expressly stated that it is their intention to authorise the payment of contributions to hospitals and to regard such payments as made on account of sickness benefit, thus carrying the advantage, so far as the approved societies are concerned, that the State allowance of two-ninths of the sum so expended (one quarter in the case of women) will be made, it is indicated, in effect, that the benefit to be derived from such contributions is so problematical that, for the moment, it is suggested that only such sums as have actually been saved on administration account should be so employed. It is true that approved societies are counselled to seek expert actuarial advice, but it is difficult to see how, without actual data, such advice can be of material assistance in determining the financial advantages that will accrue to the approved societies.

There is, fortunately, no indication in the circular that it is intended to limit the powers conferred by the Commissioners' Order, when it is issued, to the application of funds actually saved out of the sum allowed for expenses of management. If this were prescribed it is to be feared that in most cases the regulations would be without result from their inception. In the main the approved societies have experienced considerable difficulty in confining their expenses to the prescribed limits, and it is chiefly for this reason that half-yearly insurance cards are to be substituted in the New Year for the present system of quarterly cards. There are admittedly some notable exceptions among the larger approved societies, but the saving on administrative expenses has, in their case, been earmarked, as it were, as a special reserve fund to guard against a possible deficiency, as the result of adverse experience, when the first valuation is made, and as a source of profit from which additional benefits are to be granted to the members. It is therefore doubtful if these societies would be willing to entrench upon the funds so set apart.

### What Action will be Taken?

What, then, is to be the attitude of the approved societies in regard to the powers shortly to be conferred?

In our opinion it would be long-sighted policy on the part of the committees of management of the leading approved societies to take advantage of the powers in spite of the fact that no saving may have been made on account of administrative expenses, or if such saving has accrued that it has been earmarked for other purposes.

It needs no demonstration that without the hospitals the approved societies would be hopelessly insolvent. Apart from the purely financial aspect of the matter there is the advantage that would accrue to the societies of being able to rely with certainty on the services of the hospitals as in some sense a *quid pro quo* for the contributions made. As bearing on this matter an extremely interesting contribution appeared in the *Times* for Saturday last, in which the view was expressed that "the ideal arrangement would be for the hospital to be the centre for each insurance area which every doctor should feel that he could use for consultative purposes." It is acknowledged by the writer, as we remarked last week, that there is now a better feeling than formerly between the hospital authorities and the panel practitioners and the approved societies. Advantage should therefore be taken of the present situation for the ultimate benefit of insured persons generally.

NOTE.—Questions and Correspondence on all doubtful points are invited, and should be addressed to the Insurance Editor, The Hospital, 28 Southampton Street, Strand, W.C.

---

### Answers to Correspondents.

"T. R." informs us that he entered insurance in July 1912 at the age of sixty-two, paid his contributions until February 1913, when he lost employment through heart failure, and has since received sickness benefit for twenty-six weeks, thus exhausting the total amount to which he is at present entitled. He asks if the Amendment Act has affected his position, and when he will be able to claim benefit again.

Sickness benefit, as allowed under the Act, is exhausted after twenty-six weeks, and as it is necessary for at least 104 weeks to have elapsed since entry into insurance, and for at least 104 weekly contributions actually to have been paid before disablement benefit—that is to say, reduced sickness benefit of 5s. a week—can be claimed, he cannot obtain such benefit in any case until July 1914. In view of the requirement as to the payment of 104 contributions our correspondent was wise in following the suggestion of the secretary of his society by stamping his card each week himself, and he should continue to do so. If he "recovers" from his illness he will be entitled again to claim on the fund for sickness benefit on a recurrence of his incapacity, provided he is off the funds for at least twelve months. The word "recovers" is not defined in the Act, but as there is no stipulation to the contrary it would possibly be considered a recovery if the insured person is able to return to work for at least a week. There is as yet no decision on this point. If, however, our assumption is correct, and our correspondent is able to return to work and then has a recurrence of his disease, he should not put forward his claim for benefit until twelve months have expired from the last payment he actually received, that is, until August 1914. He would then be entitled under the Amendment Act, provided his contributions have been regularly paid and subject to his incapacity lasting so long, to sickness benefit, at the rate of 10s. a week, for a total period of twenty-six weeks. When this term has expired he could claim disablement benefit at the rate of 5s. a week, and this would continue until he again recovers, or until he attains the age of seventy, whichever is the longer term.

